# A laminar forced convection via transport of water–copper–aluminum hybrid nanofluid through heated deep and shallow cavity with Corcione model

**DOI:** 10.1038/s41598-023-31884-2

**Published:** 2023-03-25

**Authors:** Abid. A. Memon, M. Asif Memon, Amsalu Fenta

**Affiliations:** 1 Department of Mathematics and Social Sciences, Sukkur IBA University, Sukkur, 65200 Sindh Pakistan; 2 Department of Physics, Mizan Tepi University, PO Box 121, Tepi, Ethiopia

**Keywords:** Mechanical engineering, Materials science

## Abstract

The article explores how fluid flows and heat transfers in both deep and shallow cavities when using a nanofluid made of water, copper, and aluminum oxide. The study applies the Corcione model to hybrid nanofluids, which considers viscosity, conductivity, and the size of the nanoparticle, temperature, and Reynolds number. The cavity is connected to a rectangular channel, with the cavity's length being half the total length of the enclosure, and the aspect ratio (cavity height divided by height of the channel) is tested from 1 to 3. The study uses the Navier–Stokes equation and energy equation in two dimensions, along with finite element-based software, COMSOL 5.6, to simulate the combination of fluid flow and heat transmission. The results show a circular distribution of temperature in the cavity, and the average temperature drops as the volume fraction of copper upsurges. However, both the Reynolds number and volume fraction of copper improve the average Nusselt number, which shows how well the fluid transfers heat, along the cavity's middle line. The percentage change in the average Nusselt number decreases as the aspect ratio increases, indicating improved conduction.

## Introduction

The application of research article is in the field of heat transfer, where the use of nanofluids is gaining popularity due to their enhanced thermal conductivity compared to traditional fluids. Specifically, the present study can help engineers and researchers in the design and optimization of cooling systems, such as electronic devices, nuclear reactors, and gas turbines, where efficient heat transfer is critical for their performance and safety. The investigation of laminar forced convection through different cavity geometries is also relevant to the understanding and improvement of natural and forced convection in various engineering applications, such as heat exchangers, solar collectors, and microfluidic devices^1–4^.

In recent decades, a unique type of fluid made up of metallic particles on a nano-scale has been introduced into the market. The intention behind using this fluid is to increase the thermal conductivity of the resultant fluid, known as nanofluids, which can be utilized for heating and cooling purposes, as stated by Maxwell^5^. Numerous researchers have explored different methods to increase the thermal conductivity of nanofluids over the years. These include tactics such as increasing the volume fraction of nanoparticles in the base fluids, altering the size of metallic particles, and investigating the mixture of nanoparticles. Choi^6^ was the first to propose the idea of producing nanofluids for the purpose of improving heat transfer rates, which opened the door to further research into the thermal properties of common nanofluids. Numerous research studies have been conducted to explore the thermal conductivity of nanofluids, which has been discovered to be superior to macroscopic models. One such investigation was carried out by Eastman et al.^7^, where they examined the thermal properties of both pure base fluid and nanofluids by suspending nanoparticles in the base fluid. Their results indicated that nanofluids performed better than the base fluid in terms of heat transfer, owing to their higher thermal conductivity. In another study, Oztop et al.^8^ analyzed the heat distribution in a rectangular cavity domain using different nanofluids and discovered that increasing the Rayleigh number and the volume fraction of the nanofluids resulted in a significant improvement in heat production. The primary objective of Khanafer et al.^9^ was to evaluate the viscosity and determine the thermal conductivity of nanofluids using the Wasp model. To achieve this, they examined natural convection in a heated cavity with nanofluids, utilizing the Brinkman model^10^.

Masuda et al.^11^ attempted to improve the thermal conductivity of a mixture containing metallic particles by measuring the thermal conductivity of water alumina with a 13 nm size diameter. According to a report, the thermal conductivity of nanofluids was found to be dependent only on the volume fraction. Researchers Eastman et al.^12^ conducted a study and observed a significant increase in thermal conductivity of a mixture of water-alumina when the volume fraction was raised up to 4.3%. In another study, Mohammed et al.^13^ investigated mixed convection in a two-dimensional deep cavity under laminar flow regime conditions and found that several factors, including the volume fraction, contributed to heat transfer enhancement in the channel. Similarly, Al-Aswadi et al. also explored the effects of volume fraction on heat transfer in their research. Al-Aswadi et al.^14^ conducted a forced convection study using nanofluids in the backward step cavity and reported that the reattachment length and vortices at the corner both increased with the increase in Reynolds number.

Xuan and Li^15^ investigated a forced convection problem in the turbulent regime using water-copper nanofluids and found that they substantially enhanced the heat transfer rate when compared with the pure base fluid. Kalth et al.^16^ conducted an experiment on forced convection heat transfer through a small rectangular channel with a heat sink using water-alumina nanofluid under fixed heat flux conditions. They reported that only the mixture containing the 0.1% and 0.2% volume fraction is sufficient to enhance the heat transfer rate, and also increment of heat transfer rate plays a vital role in heat production. In another study, Kherbeet et al.^17^ developed a simulation for mixed convection through a backward step channel in both 2D and 3D spaces. The results revealed that increasing the step height of the backward stepping channel increased both the skin friction coefficient and the average Nusselt number, while the Reynolds number and pressure drop declined. Nie and Armaly^18^ developed a simulation to investigate forced convection heat transfer through a three-dimensional backward-facing step channel in a rectangular cavity. The results showed that the primary vortex beside the corner increased by increasing the step height of the channel. It was also explained that an increase in the step height enhances the maximum average Nusselt number. Further related work can be found in references^19–21^.

The aim of this investigation is to examine the behavior of a rectangular channel that is linked to both a deep and shallow cavity. A hybrid nanofluid comprising copper and aluminum oxide will be transported through the channel. The cavity will have a base that is half the length of the rectangular channel, and the initial height of the cavity will be the same as that of the rectangular channel. The aspect ratio, which is the ratio of the cavity height to the rectangular channel height (with fixed channel height), will be altered from 1 to 3. The research will focus on forced convection through laminar flow, with Reynolds numbers ranging from 100 to 1000. The volume fraction of copper-alumina in the base fluid will vary from 0.01 to 0.11, and the resulting viscosity and thermal conductivity will be determined using the Corcione model. The study will present findings for several variables, including the temperature distribution, percentage variation in the center of the cavity, minimum temperature along the middle of the cavity, average Nusselt number, and the Darcy–Weisbach friction force on the domain.

## Problem formulation

**Figure 1:**
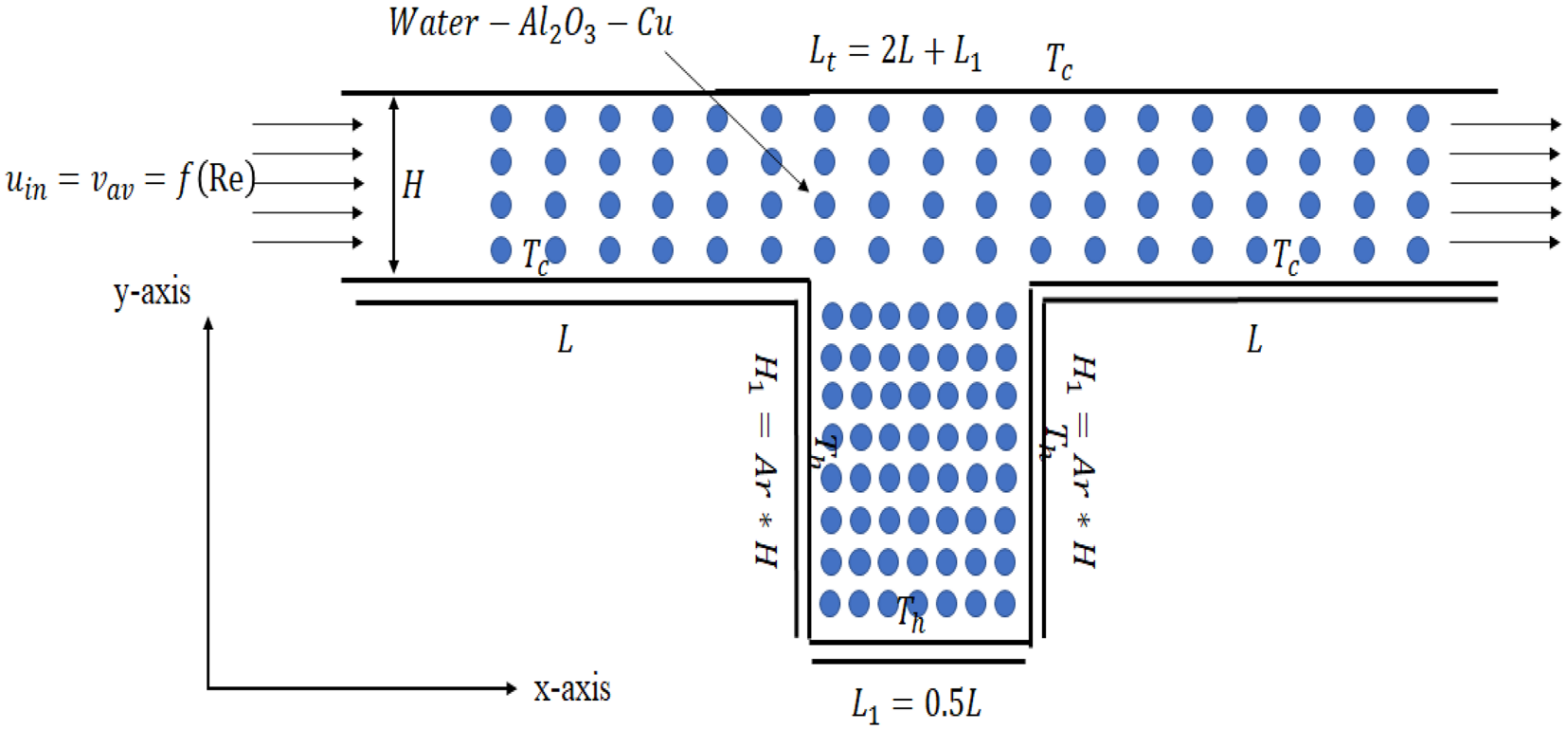
A view of the two-dimensional rectangular channel with a deep cavity attached at the middle of the channel.

In Fig. 1, let H represent the height of the channel and L represent the length of the mainstream before the channel cavity. For the upcoming problems 1–3, Ar will be the aspect ratio between the channel height and the cavity height, with intervals of 0.5, while H_1_ will be the height of the cavity. Additionally, L_1_ is half the length of the mainstream, as shown in Fig. 1. To study forced convection, a mixture of Alumina (Al_2_O_3_) and copper (Cu) with water will be introduced into the channel from the left entrance, with an initial velocity u_in_. The Reynolds number will fall within a range of 100–1000, and the proportion of copper oxide to aluminum oxide in water will be twice as much for the former. To study convection, the inner walls of the cavity will be subjected to a high temperature, while the exterior of the cavity will be maintained at a lower temperature. The Corcione et al.^22^ model will be used to simulate the transport of the hybrid mixture. The thermal conductivity of the hybrid mixture is reliant on temperature, while the viscosity is affected by the diameter size of the nanoparticles present in the nanofluids. This is a key benefit of the model. Table 1 provides the parameters of the geometry and empirical equations for the hybrid mixture, while Table 2 displays the computational procedure of COMSOL 5.6 used in the simulation.

**Table 1:** Parameters and thermo-physical properties of the hybrid nanofluids ^22^.

H	10 [cm]	Height of the channel
L	40 [cm]	Length of the channel
H_1_	Ar (H)	Depth of the cavity
Ar	1, 1.5, 2, 2.5, 3	The aspect ratio of inlet height to the height of the cavity
L_1_	20 [cm]	Height of the obstacle
$T_{c}$	293 [K]	The cold temperature along the wavy
$T_{h}$	313 [K]	The hot temperature along the obstacle
$\kappa_{{H_{2} O}}$	0.628 [W/(mK)]	Thermal conductivity of water
$\kappa_{Cu}$	400 [W/(mK)]	Thermal conductivity of copper
$\kappa_{{Al_{2} O_{3} }}$	40 [W/(mK)]	Thermal conductivity of the aluminum oxide
$\mu_{{H_{2} O}}$	0.000695 [Pas]	Viscosity of water
$\rho_{{H_{2} O}}$	993 [kg/m^3^]	Density of water
$\rho_{Cu}$	8933 [kg/m^3^]	Density of copper
$\rho_{{Al_{2} O_{3} }}$	3970 [kg/m^3^]	The density of the aluminum oxide
$(c_{p} )_{{H_{2} O}}$	4178 [J/(kgK)]	Heat capacity at constant pressure
$(c_{p} )_{Cu}$	385 [J/(kgK)]	Heat capacity of copper
$(c_{p} )_{{Al_{2} O_{3} }}$	765 [J/(kgK)]	Heat capacity of aluminum oxide
$(d_{p} )_{{H_{2} O}}$	0.385 [nm]	Particle's diameter in the water
$(d_{p} )_{Cu}$	29 [nm]	Particles diameter of copper
$(d_{p} )_{{Al_{2} O_{3} }}$	33 [nm]	Particles diameter of aluminum oxide
$\phi_{Cu}$	0.01, 0.05, 0.09, 0.11	The volume fraction of copper
$\phi_{{Al_{2} O_{3} }}$	2 $\phi_{Cu}$	The volume fraction of aluminum oxide
$(\rho c_{p} )_{hnf}$	$$\begin{gathered} \phi_{Cu} \rho_{Cu} (c_{p} )_{Cu} + \phi_{{Al_{2} O_{3} }} \rho_{{Al_{2} O_{3} }} (c_{p} )_{{Al_{2} O_{3} }} \hfill \\ + (1 - \phi_{Cu} - \phi_{{Al_{2} O_{3} }} )\rho_{{H_{2} O}} (c_{p} )_{{H_{2} O}} \hfill \\ \end{gathered}$$	Heat capacitance of hybrid nanofluid
$\rho_{hnf}$	$\phi_{Cu} \rho_{Cu} + \phi_{{Al_{2} O_{3} }} \rho_{{Al_{2} O_{3} }} + (1 - \phi_{Cu} - \phi_{{Al_{2} O_{3} }} )\rho_{{H_{2} O}}$	The density of the hybrid mixture
$\mu_{hnf}$	$\frac{{\mu_{{H_{2} O}} }}{{1 - 34.87(d_{f} )_{{H_{2} O}} [((d_{p} )_{Cu} )^{0.3} (\phi_{Cu} )^{1.03} + ((d_{p} )_{{Al_{2} O_{3} }} )^{0.3} (\phi_{{Al_{2} O_{3} }} )^{1.03} ]}}$	The viscosity of the hybrid mixture
M	18 [g/mol]	The molecular weight of water
$d_{f}$	$0.1\left( {\frac{6M}{{N_{A} \pi \rho_{{H_{2} O}} }}} \right)$	Molecular diameter of the water
$\Pr$	$\frac{{\mu_{{H_{2} O}} }}{{(cp)_{{H_{2} O}} \kappa_{{H_{2} O}} }}$	Prandtl number
$T_{fr}$	273.2 [K]	The freezing temperature of the water
$u_{in}$	$\frac{{{\text{Re}} \mu_{hnf} }}{{\rho_{hnf} D_{h} }}$	Inlet velocity of hybrid mixture
A	$L_{t} H + L_{1} H_{1}$	The total area of the channel
$P_{r}$	$2\left( {L_{t} + H + H_{1} } \right)$	The total perimeter of the channel
$D_{h}$	$\frac{4A}{{P_{r} }}$	Hydraulic Diameter
Re	100–1000	Reynolds number
$u_{b}$	$\frac{{2k_{b} T}}{{\pi \mu_{{H_{2} O}} \left[ {\left( {d_{p} } \right)_{Cu} + \left( {d_{p} } \right)_{{Al_{2} O_{3} }} } \right]}}$	The Brownian velocity of nanoparticles
${\text{Re}}_{B}$	$\frac{{\rho_{{H_{2} O}} u_{b} \left[ {\left( {d_{p} } \right)_{Cu} + \left( {d_{p} } \right)_{CU} } \right]}}{{\mu_{{H_{2} O}} }}$	Reynolds number for nanoparticles
$\frac{{\kappa_{hnf} }}{{\kappa_{{H_{2} O}} }}$	$$\begin{gathered} 1 + 4.4{\text{Re}}_{b}^{0.4} \Pr^{0.66} \left( {\frac{T}{{T_{fr} }}} \right)^{10} \left( {\kappa_{{H_{2} O}} } \right)^{ - 0.03} \left[ {\left( {\kappa_{Cu} } \right)^{0.03} \left( {\phi_{Cu} } \right)^{0.66} } \right. \hfill \\ \left. { + \left( {\kappa_{{Al_{2} O_{3} }} } \right)^{0.03} \left( {\phi_{{Al_{2} O_{3} }} } \right)^{0.66} } \right] \hfill \\ \end{gathered}$$	Thermal conductivity of the hybrid nanofluid

**Table 2:** The working Wagon Wheel of COMSOL 5.6.

Step 1: Select the dimensions of the geometry which can be updated with just a click and which is changeable for parametric study
Step 2: Selecting the parameters to impose the material for forced convection for example hybrid nanofluids are imposed here so that all hermos physical properties of hybrid nanofluids are selected like a Table 1
Step 3: Utilize the graphical interface of COMSOL 5.6 to create the channel's geometry
Step 4: Imposing material by selecting the geometry
Step 5: Applying the feasible boundary condition for Laminar flow and heat transfer
Step 6: Mesh or grid-independent test to ensure a better degree of accuracy than the previous solution. Then, validating the results with previous publishing literature
Step 7: Post-processing procedure using the derived values option or line plots

It has been decades since the Navier–Stokes equations are serving in the field of fluid dynamics to understand the dynamics of fluid particles in a certain domain of interest. A deep cavity situated within a rectangular channel will undergo simulation using the finite element package COMSOL Multiphysics 5.6 to investigate the transport of a hybrid mixture. In simulating the present problem, the incompressible Navier Stokes equations will be considered along with the convection–diffusion Eq. (1)–(4). The general description of the boundary condition for the current problem is given as (5)–(9). The formula to compute the numerical results is given as (10)–(16).

### Governing equations and boundary equations

1 $$ \frac{\partial u}{{\partial x}} + \frac{\partial v}{{\partial x}} = 0 $$

2 $$ u\frac{\partial u}{{\partial x}} + v\frac{\partial u}{{\partial x}} + \frac{1}{{\rho_{hnf} }}\frac{\partial p}{{\partial x}} - \frac{{\mu_{hnf} }}{{\rho_{hnf} }}\left( {\frac{{\partial^{2} u}}{{\partial x^{2} }} + \frac{{\partial^{2} u}}{{\partial y^{2} }}} \right) = 0 $$

3 $$ u\frac{\partial v}{{\partial x}} + v\frac{\partial v}{{\partial x}} + \frac{1}{{\rho_{hnf} }}\frac{\partial p}{{\partial y}} - \frac{{\mu_{hnf} }}{{\rho_{hnf} }}\left( {\frac{{\partial^{2} v}}{{\partial x^{2} }} + \frac{{\partial^{2} v}}{{\partial y^{2} }}} \right) = 0 $$

4 $$ u\frac{\partial T}{{\partial x}} + v\frac{\partial T}{{\partial x}} - \frac{{\kappa_{hnf} }}{{\rho_{hnf} (c_{p} )_{hnf} }}\left( {\frac{{\partial^{2} T}}{{\partial x^{2} }} + \frac{{\partial^{2} T}}{{\partial y^{2} }}} \right) = 0 $$

### Boundary conditions

5 $$ At\,Inlet: u = u_{in,} v = 0,\frac{\partial T}{{\partial n}} = 0 \,at\, x = 0, 0 \le y \le H $$

6 $$ At \,outlet: u,v \ne 0,\frac{\partial T}{{\partial n}} = 0 \,at\, x = L_{t} , 0 \le y \le H $$

7 $$ At\, the\, top\, surface: u = v = 0, T = T_{c} \,when\, y = H, 0 \le x \le L_{t} $$

8 $$ At\, main\, stream: u = v = 0, T = T_{c} = \left\{ \begin{gathered} y = 0, 0 \le x \le L \hfill \\ y = 0, L_{1} + L \le x \le L_{t} \hfill \\ \end{gathered} \right. $$

9 $$ In\, the\, heated\, cavity: u = v = 0, T = T_{h} = \left\{ \begin{gathered} x = L, - H_{1} \le y \le 0 \hfill \\ y = - H_{1} , L \le x \le L + L_{1} \hfill \\ x = L + L_{1} , - H_{1} \le y \le 0 \hfill \\ \end{gathered} \right. $$

### Computational parameters

10 $$ {\text{Heat}}\;{\text{Source}}:Q = \kappa_{hnf} \nabla T $$

11 $$ {\text{Coefficient}}\;{\text{of}}\;{\text{convection}}\;{\text{heat}}\;{\text{transfer}}:h = \frac{Q}{{A(T - T_{b} )}} $$

12 $$ {\text{Bulk}}\;{\text{Temperature}}:T_{b} = \frac{{T_{c} + T_{b} }}{2} $$

13 $$ {\text{Local}}\;{\text{Nusselt}}\;{\text{number}}\;{\text{along}}\;{\text{the}}\;{\text{x}}\;{\text{or}}\;{\text{y}}\;{\text{direction}}:Nu_{x} = \frac{hx}{{\kappa_{hnf} }},Nu_{y} = \frac{hy}{{\kappa_{hnf} }} $$

14 $$ {\text{Average}}\;{\text{Nusselt}}\;{\text{number}}:(Nu_{x} )_{avg} = \int {Nu_{x} dx} ,(Nu_{y} )_{avg} = \int {Nu_{y} dx} $$

15 $$ {\text{Darcy - Weisbach}}\;{\text{equation}}\;{\text{of}}\;{\text{friction}}\;{\text{factor}}:f_{D} = \frac{{2D_{h} \Delta P}}{{\rho_{hnf} V_{mean}^{2} L}} $$

16 $$ {\text{Percentage}}\;{\text{change}}\;{\text{due}}\;{\text{to}}\;{\text{cold}}\;{\text{temperature}}:\Delta T_{c} = 100\left( {\frac{{T - T_{c} }}{{T_{c} }}} \right) $$

## Mesh independent study and validation of the code

**Figure 2:**
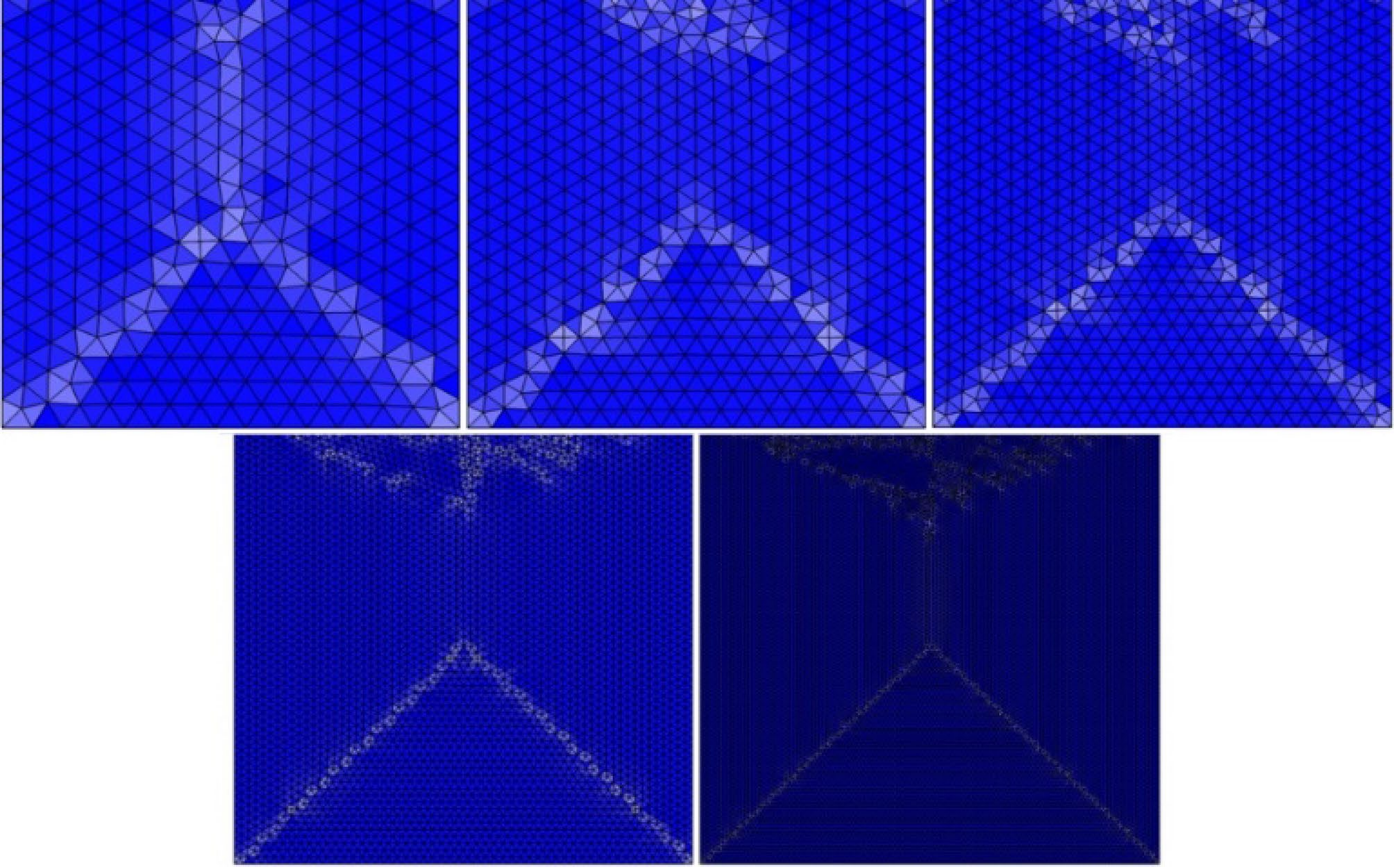
The meshing process in the cavity from coarse to extremely fine mesh.

**Figure 3:**
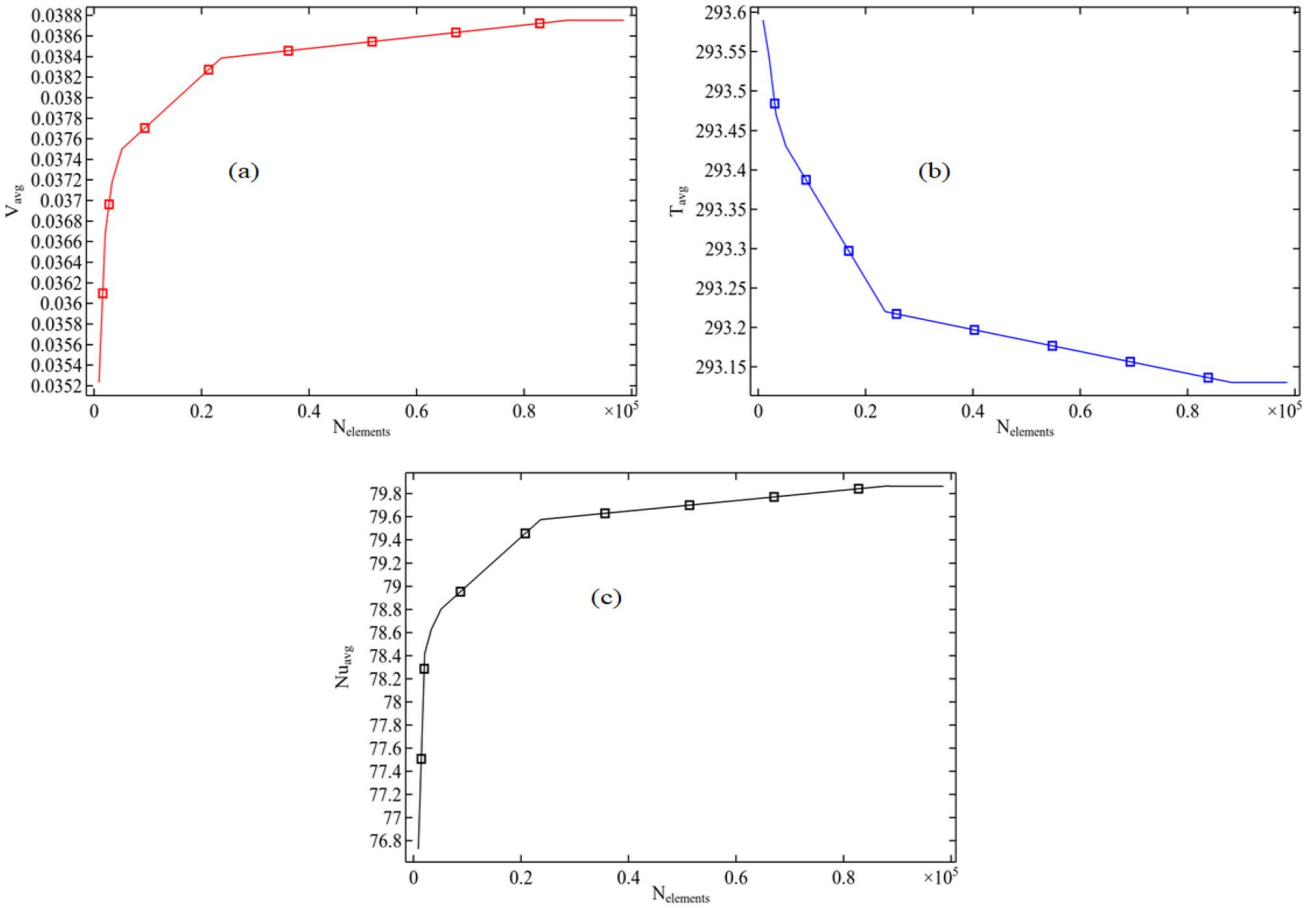
Mesh independent study for the numerical results calculated in the full domain for (**a**) Average velocity (**b**) Average Temperature and (**c**) Average Nusselt number.

To conduct the mesh independency test, three variables are selected. Figure 2 displays the meshing procedure used for the deep cavity of the channel, where the geometry is meshed with irregular triangular elements. For each number of elements, we determine the numerical outcome for the selected three variables. Figure 3a–c displays the computational outcomes of the average velocity, temperature, and Nusselt number for the entire channel domain as the element count gradually increases to create a dense mesh. This is done to guarantee superior numerical results. As the number of elements increases, the precision of each variable improves, as indicated by the results. The numerical approach attains a mesh-independent solution beyond 80,000 elements. For this problem, we simulated using approximately 88,000 irregular triangular elements.

**Figure 4:**
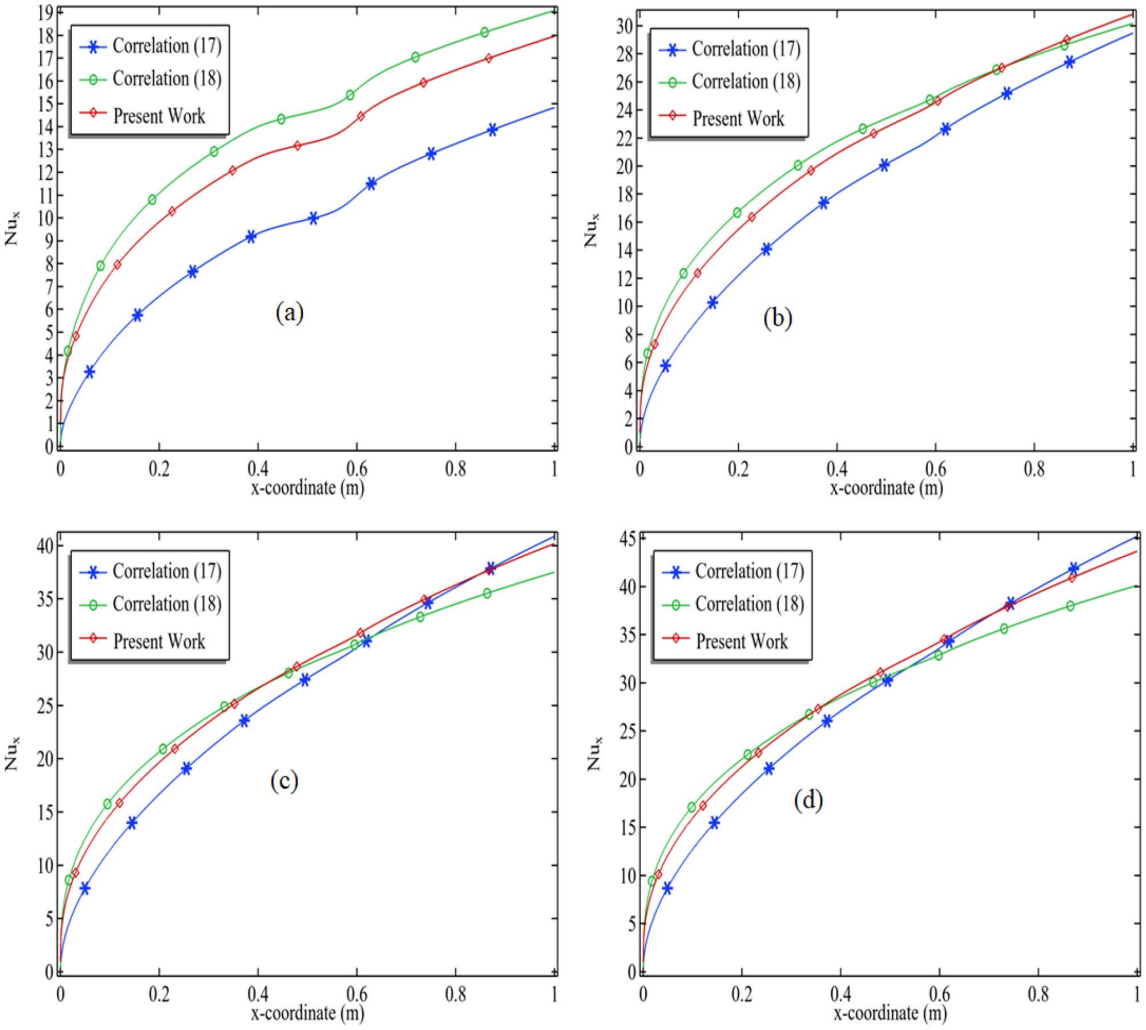
Validation of the code by calculating the local Nusselt number at the position y = H/2 when (**a**) Re = 100 (**b**) Re = 400 (**c**) Re = 800 and (**d**) Re = 1000.

The validation of numerical results for the local Nusselt number involves comparing them with two correlations (17) and (18)^23,24^. The position y = H/2 along the middle line of the rectangular channel is used to calculate the local Nusselt number for Reynolds numbers Re = 100, 400, 800, 1000, as shown in Fig. 4a–d. The comparison shows a close relationship between the present work and the correlations (17) and (18). Thus, the present code can be deemed reliable in generating numerical results. Figure 4a–d demonstrates that the present work gets closer to experimental correlations as the Reynolds number increases.

17 $$Nu_{x}  = 0.332\text{Re} _{x}^{{1/2}} {\Pr}^{{1/3}}$$

18 $$ Nu_{x} = 1.62{\text{Re}}_{x} \Pr \left( {\frac{{D_{h} }}{L}} \right)^{1/3} $$

**Figure 5:**
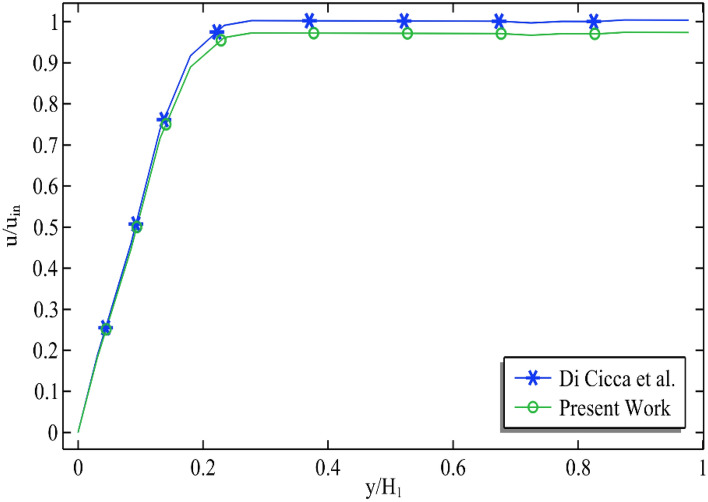
Comparison of the non-dimensional velocity profile near the age of the cavity against the non-dimensional with different works when material is water ($\phi_{Cu} = \phi_{{Al_{2} O_{3} }} = 0$).

We are also trying to compare the present work with previously published literature^25^ by establishing a velocity profile at the edge of the cavity against the non-dimensional length see Fig. 5. It can be seen that the present code is working well with the previous publishing literature.

## Results and discussion

### Temperature distribution in the cavity

**Figure 6:**
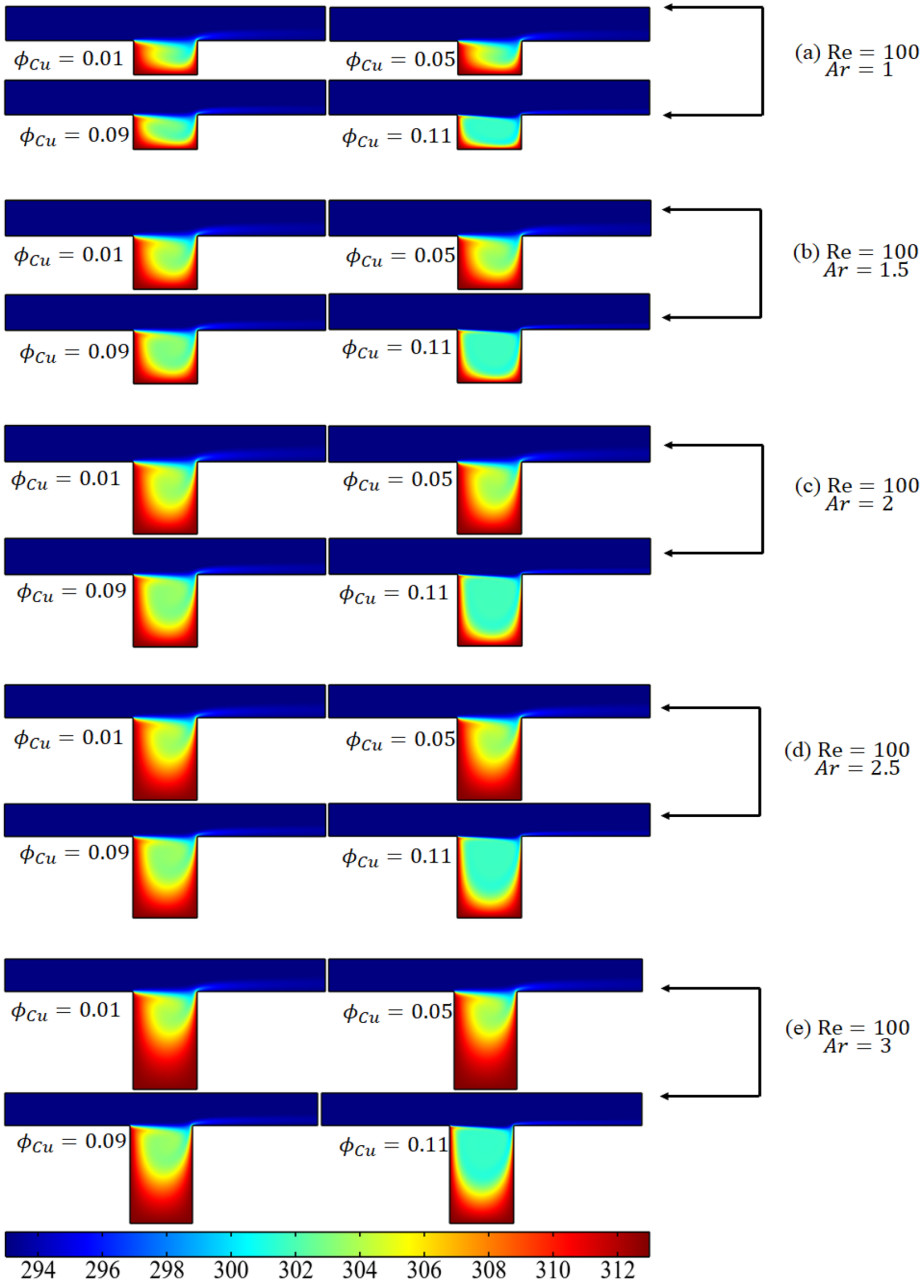
The temperature distribution in the cavity when Re = 100.

This passage examines the outcomes of a simulation that investigated the behavior of convection–diffusion within a rectangular cavity. The simulation entailed introducing a cold temperature outside the cavity and a hot temperature inside to gauge the resulting effects. The results of this simulation are presented in Fig. 6a–e, where the temperature distribution is shown through surface plots. The Reynolds number was fixed at 100 and the aspect ratio was between 1 and 3, while the volume fraction of copper was altered for each case. In Fig. 6a, it can be seen that when the hybrid mixture entered the cavity, the average temperature in the cavity decreased due to the hot temperature imposed in the channel. Increasing the volume fraction of copper caused the temperature inside the cavity to decrease even further. The impact of this coldness was felt up to the center of the cavity. As the height of the cavity was increased (see Fig. 6b–e), a circular distribution of the temperature could be seen. It was also concluded that increasing the volume fraction of the cold fluid caused the temperature of the hot cavity to decrease compared to the surroundings. Overall, the simulation demonstrated how different variables can impact the temperature distribution within a cavity.

**Figure 7:**
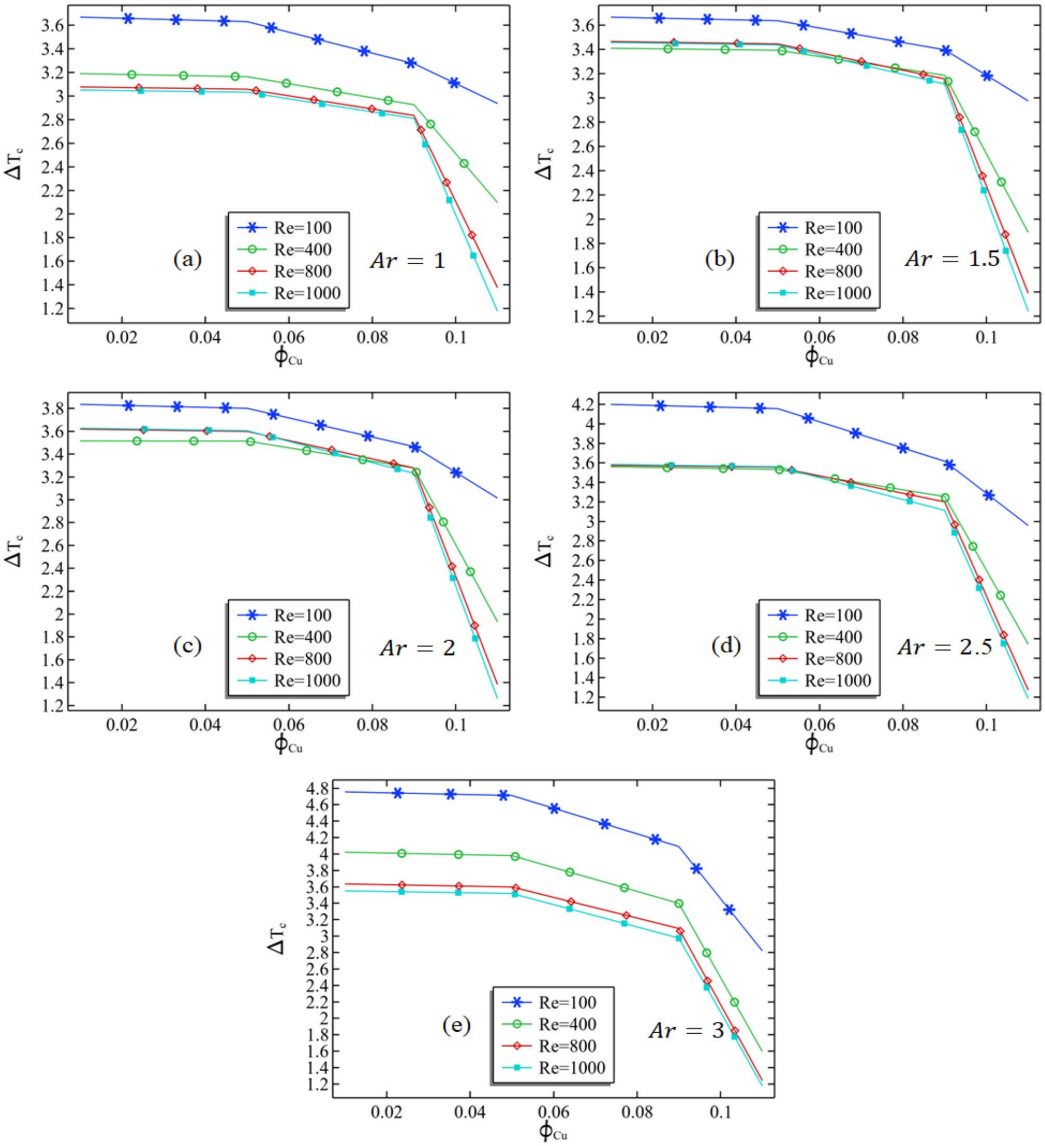
The percentage change in the temperature due to the cold temperature against the increase in the volume fraction of copper at the center of the cavity.

The analysis of Fig. 6a–e indicates that an increase in the copper volume fraction leads to a decrease in the average temperature. In contrast, Fig. 7a–e investigates the percentage shift in temperature at the cavity's core caused by the introduction of a cold temperature. A specific location (L + L_1_/2, − H_1_/2) was chosen to examine the effect of varying the volume fraction of copper, Reynolds number, and aspect ratio on the percentage change due to the cold temperature. The graph in Fig. 7a illustrates that at a constant aspect ratio and Reynolds number, the reduction in temperature caused by the lower temperature is lessened as the volume percentage of copper is increased. Specifically, when Re = 100 and Ar = 1, the percentage change in temperature decreases from 3.65 to 3 when the volume fraction of copper is increased from 0.01 to 0.11.The figure also indicates that an increase in Reynolds number further decreases the percentage change in temperature due to the cold temperature. This can be attributed to the fact that increasing the inlet velocity of the fluid results in more cold fluid entering the cavity, thus decreasing the temperature. Additionally, as the height of the cavity is increased, the percentage change in temperature increases as the center of the channel moves further away. The percentage change in temperature generally decreases for volume fractions between 0.01 and 0.09 of the volume fraction of copper at a fixed Reynolds number, and abruptly drops when the volume fraction exceeds 0.09, as shown in Fig. 7e.

Table 3 presents the minimum temperature along the middle line of the cavity. Analyzing the table reveals that, for a constant volume fraction and Reynolds number, increasing the aspect ratio of the cavity results in higher minimum temperatures. On the other hand, for a particular Reynolds number and aspect ratio, increasing the volume fraction of copper decreases the minimum temperature. Additionally, an elevation in Reynolds number results in a positive response from the minimum temperature. After analyzing the data provided in Table 3, it can be inferred that the most favorable minimum temperature along the cavity's central axis is attained at a Reynolds number of 100 and aspect ratio of 1.

**Table 3:** Minimum temperature $T_{\min }$ at the middle of the cavity for all parameters used to develop the simulation.

Re	$\phi_{Cu}$	Ar = 1	Ar = 1.5	Ar = 2	Ar = 2.5	Ar = 3
100	0.01	296.8	297.34	297.35	297.27	297.18
100	0.05	296.71	297.26	297.27	297.19	297.1
100	0.09	295.56	296.17	296.17	296.08	295.97
100	0.11	293.13	293.4	293.36	293.33	293.28
400	0.01	299.29	299.82	299.81	299.79	299.76
400	0.05	299.26	299.8	299.82	299.77	299.73
400	0.09	298.95	299.56	299.51	299.41	299.36
400	0.11	297.58	297.91	297.53	296.95	296.81
800	0.01	299.72	300.55	300.45	300.33	300.37
800	0.05	299.71	300.54	300.46	300.31	300.34
800	0.09	299.51	300.38	300.22	299.96	299.91
800	0.11	296.98	296.95	296.86	296.39	296.4
1000	0.01	299.78	300.72	300.62	300.46	300.45
1000	0.05	299.78	300.72	300.61	300.43	300.44
1000	0.09	299.57	300.57	300.38	299.99	299.91
1000	0.11	296.43	296.54	296.6	296.23	296.27

Physical explanation of results above: When the volume fraction of copper is increased, it results in a corresponding increase in the thermal conductivity of the fluid within the cavity. This increase in thermal conductivity facilitates the transfer of heat from the cavity to the copper, making it more efficient. Consequently, the average temperature within the cavity decreases. The temperature distribution within the cavity is circular and is a result of the interaction between the fluid flow within the cavity and the cold temperature imposed within. As the fluid flows towards the center of the cavity, it is exposed to the cold temperature, leading to a temperature decrease at the center. By increasing the aspect ratio of the cavity, more efficient heat transfer from the cavity to the environment is possible, leading to an increase in the minimum temperature along the middle line of the cavity. Meanwhile, an increase in the volume fraction of copper leads to an increase in the thermal conductivity of the fluid inside the cavity. This increase in thermal conductivity enables a more efficient transfer of heat from the cavity to the copper, resulting in a decrease in the minimum temperature..

### Average Nusselt number at the middle line of the cavity

**Figure 8:**
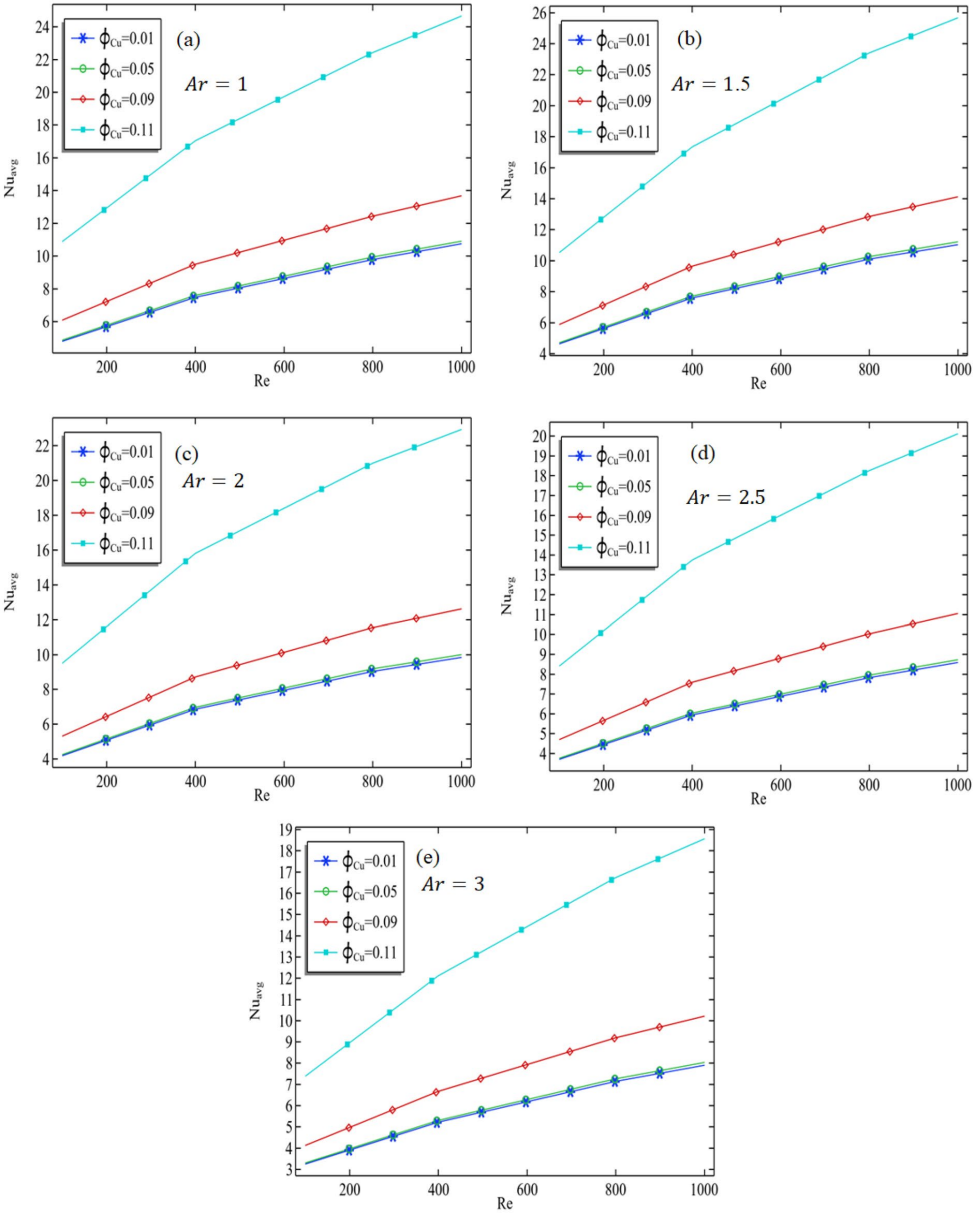
The average Nusselt number at the middle of the cavity against increasing Reynolds number with different aspect ratios and volume fraction.

The Nusselt number represents the ratio of convection to conduction processes and is a function of Prandtl and Reynolds numbers. A higher Nusselt number indicates that convection is occurring at a faster rate than conduction, or vice versa. Figure 8a–e exhibit the mean Nusselt number along the central axis of the cavity at constant volume fraction and aspect ratio as Reynolds number increases. As depicted in Fig. 8a, the average Nusselt number escalates with the rise in Reynolds number, suggesting that convection outpaces conduction. The reason behind this is that a greater Reynolds number allows a larger amount of nanofluid to enter the cavity. Additionally, the average Nusselt number increases as the volume fraction of copper increases, given a fixed aspect ratio. Table 4 shows that the average Nusselt number increases along the middle line of the cavity as the aspect ratio increases from 1 to 1.5 but then decreases when it increases from 1.5 to 3. Although there is only a slight increase in the Nusselt number as the volume fraction is increased by altering the aspect ratio of the cavity, it can be concluded that the convection process is mainly affected by an increase in the aspect ratio of the cavity, which can be compensated for by increasing the Reynolds number.

**Table 4:** The average Nusselt number along the middle line passing through the cavity of the channel.

Re	$\phi_{Cu}$	$Nu1_{avg} \%$	$Nu2_{avg} \%$	$Nu3_{avg} \%$	$Nu4_{avg} \%$
100	0.01	− 3.34383	− 9.69266	− 11.5259	− 12.2176
100	0.05	− 3.34353	− 9.69167	− 11.5289	− 12.2179
100	0.09	− 3.34362	− 9.69145	− 11.5292	− 12.2172
100	0.11	− 3.33425	− 9.67313	− 11.5106	− 12.2043
400	0.01	1.482669	− 9.65318	− 13.4118	− 12.0872
400	0.05	1.482115	− 9.6492	− 13.4264	− 12.0742
400	0.09	1.499321	− 9.63014	− 12.9115	− 12.0558
400	0.11	1.748621	− 8.812	− 13.0597	− 11.8571
800	0.01	3.198921	− 10.5631	− 13.3921	− 8.69737
800	0.05	3.2007	− 10.4813	− 13.4664	− 8.60367
800	0.09	3.320203	− 10.0996	− 13.1556	− 8.34164
800	0.11	4.374387	− 10.2918	− 13.0441	− 8.33014
1000	0.01	2.594625	− 10.7777	− 12.7217	− 8.00158
1000	0.05	2.832783	− 10.8318	− 12.7255	− 7.94345
1000	0.09	3.230994	− 10.6359	− 12.4247	− 7.58234
1000	0.11	4.108867	− 10.6362	− 12.3033	− 7.71066

Here

*Nu*1_*avg*_% = The percentage change in the average Nusselt number when the aspect ratio is turned from 1 to 1.5

*Nu*2_*avg*_% = The percentage change in the average Nusselt number when the aspect ratio is turned from 1.5 to 2

*Nu*3_*avg*_% = The percentage change in the average Nusselt number when the aspect ratio is turned from 2 to 2.5

*Nu*4_*avg*_% = The percentage change in the average Nusselt number when the aspect ratio is turned from 2.5 to 3.

Physical explanation of results above: These findings may be explained by variations in the flow pattern and the creation of boundary layers inside the cavity. When the aspect ratio is lower, the flow within the cavity is likely to be more intricate, leading to a rise in mixing and convective heat transfer. However, when the aspect ratio is higher, the flow becomes less active, and the development of boundary layers can impede heat transfer. Furthermore, changes in aspect ratio can influence the alignment of the cavity with respect to gravity's direction, leading to modifications in the flow pattern and heat transfer.

### Darcy–Weisbach friction factor

A well-known formula used for finding the dimensionless friction factor coefficient is the Darcy–Weisbach equation. This empirical equation incorporates several factors such as pressure loss, fluid density, hydraulic diameter, pipe length, and average velocity in the pipe. Currently, it is considered the most reputable equation for calculating the friction factor, and it is frequently used in conjunction with the Moody diagram^26^ and other empirical formulas.

**Figure 9:**
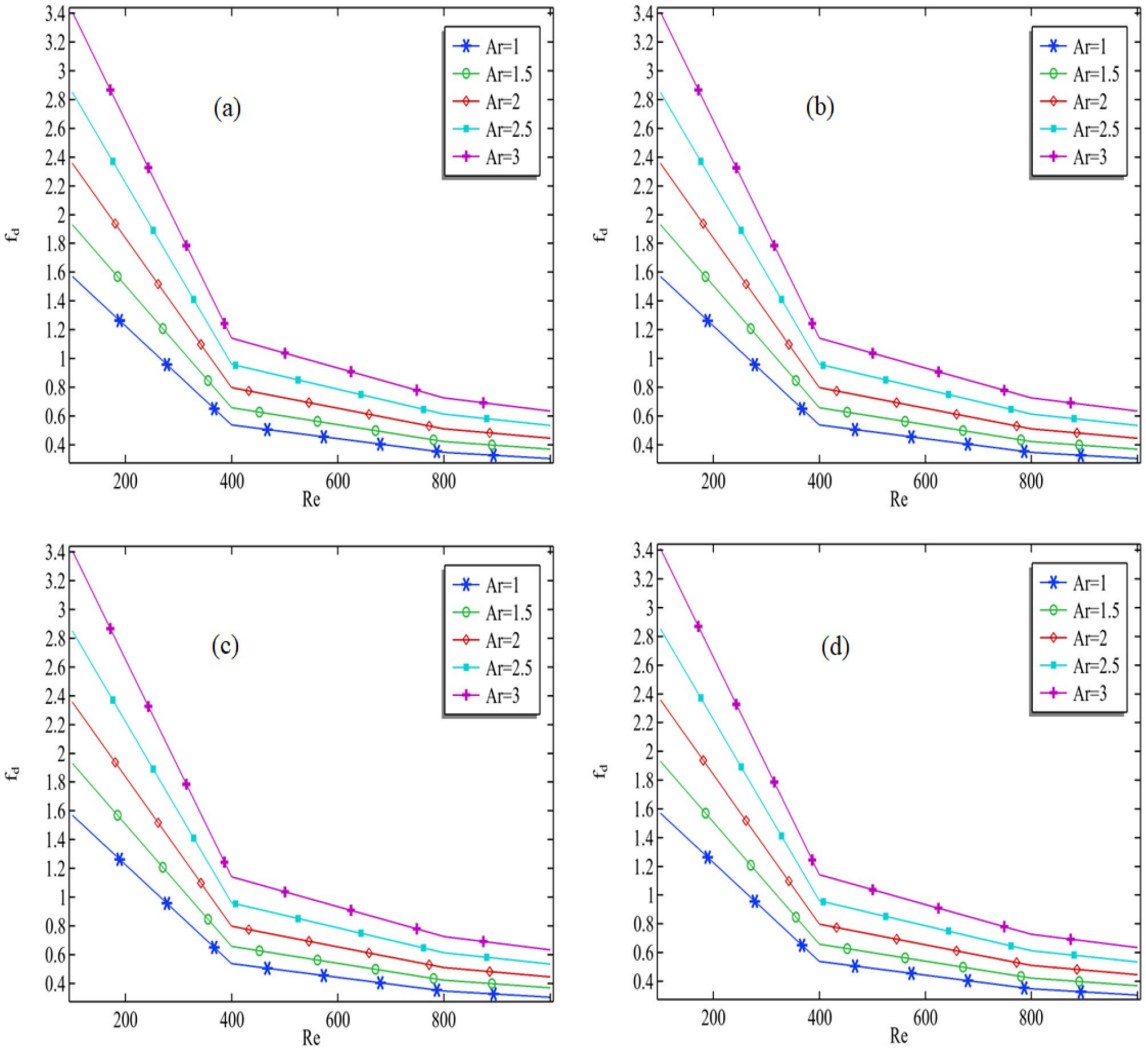
The numerical results for the Darcy–Welsbach friction factor in the whole domain.

The objective is to determine the Darcy–Weisbach friction factor in the domain and to investigate the impact of various selected parameters. Figure 9a–d depict the friction factor against Reynolds number while fixing the aspect ratio for each graph. With an increase in Reynolds number, the Darcy friction factor decreases. This decrease is due to the decrement in the viscosity of nanofluids, which in turn results in an increase in the initial and average flow velocity. Hence, according to Eq. (15), the friction factor is inversely proportional to the Reynolds number. As shown in Fig. 9a, the friction factor increases as the aspect ratio increases. This is likely because an increase in the domain enhances the hydraulic diameter, which, as per Eq. (15), is directly proportional to the friction factor. Thus, an increase in hydraulic diameter supports the friction factor to increase in the domain.

However, from the graph, it can be seen that an increase in the volume fraction of copper does not influence the Darcy friction factor in the whole domain, which might be wrong. To understand the better impact of volume friction in the Darcy friction Table 5 is given. In Table 5, we are computing the percentage change in the friction factor when the aspect ratio from the first value to the next. It can be seen that for Re = 100 the percentage change is negligible when the volume fractions of copper are changing from 0.01 to 0.09, but the major impact only can be seen when the volume fraction is changing from 0.09 to 0.11. Also, this impact can be seen for Re = 400–1000. Therefore, by increasing the aspect ratio the Darcy–Weisbach friction factor is increased, but the values of Reynolds must be sufficient.

**Table 5:** Percentage change in the friction force when the aspect ratio of the cavity is changed to the next parameter.

Re	$\phi_{Cu}$	$f_{{D_{1} }} \%$	$f_{{D_{2} }} \%$	$f_{D3} \%$	$f_{D4} \%$
100	0.01	22.97891	22.10423	20.95796	19.56087
100	0.05	22.97891	22.10423	20.95796	19.56087
100	0.09	22.97891	22.10423	20.9622	19.56369
100	0.11	22.99854	22.11578	20.98122	19.58922
400	0.01	22.30869	21.31158	20.34583	18.94981
400	0.05	22.30973	21.31212	20.32348	18.9555
400	0.09	22.33017	21.30903	20.35016	18.91413
400	0.11	22.34369	21.14581	20.43527	18.88221
800	0.01	21.65719	20.94987	20.10239	18.41164
800	0.05	21.70212	20.90062	20.1471	18.34892
800	0.09	21.67493	20.81911	20.23697	18.36292
800	0.11	21.49212	20.92764	20.06742	18.62492
1000	0.01	21.50846	20.71793	20.03464	18.74637
1000	0.05	21.45425	20.73254	20.03508	18.69379
1000	0.09	21.60301	20.60545	20.05798	18.48691
1000	0.11	21.41847	20.72338	19.97576	18.75047

Where

$f_{{D_{1} }} \%$ = The percentage when the aspect ratio is turned from 1 to 1.5.

$f_{{D_{2} }} \%$ = The percentage when the aspect ratio is turned from 1.5 to 2.

$f_{{D_{3} }} \%$ = The percentage when the aspect ratio is turned from 2 to 2.5.

$f_{{D_{4} }} \%$ = The percentage when the aspect ratio is turned from 2.5 to 3.

Physical explanation of the results above: The results explained in the preceding section are supported by several factors affecting the friction factor, including the Reynolds number, aspect ratio, and volume fraction of copper. An increase in Reynolds number results in a lower friction factor, whereas an increase in aspect ratio and hydraulic diameter supports a higher friction factor. In the domain, the volume fraction of copper has a negligible effect on the friction factor, except when the volume fraction varies from 0.09 to 0.11.

## Conclusion

The research examined various factors, including the temperature distribution, local and average Nusselt number, and Darcy–Weisbach friction factor, in a deep cavity that was secured by a rectangular channel. A hybrid mixture of copper and aluminum oxide was used, with the volume fraction of copper being double that of aluminum oxide. Forced convection was induced through the channel by adjusting the Reynolds number between 100 and 1000. Additionally, the aspect ratio was modified to explore the influence of heat distribution within the cavity, with the rectangular channel's height being compared to the cavity's height. COMSOL Multiphysics 5.6 software was utilized to discretize the incompressible Navier–Stokes and energy equations using finite element code. The Corcione model was employed to determine the empirical equations for viscosity and density of the hybrid mixture, which takes into account factors such as the Brownian velocity of the nanoparticles and the size of the nanoparticle's diameter. The research yielded several noteworthy findings.

- For a constant Reynolds number and aspect ratio, the average temperature in the heated deep cavity decreases with increasing volume fraction of copper. A circular distribution of temperature was observed in the deep cavity.
- The percentage change is due to the cold temperature at the middle or center of the cavity, and the minimum temperature in the cavity also decreases with increasing volume fraction and Reynolds number, while increasing with aspect ratio.
- When the Reynolds number and volume fraction of copper increase, the average Nusselt number along the middle line of the cavity also increases. This implies that higher convection is favored by these factors. Conversely, if the aspect ratio is increased, the average Nusselt number along the middle line decreases, assuming the volume fraction and Reynolds number remain constant.
- When the aspect ratio is changed from 1 to 1.5, the percentage change in the average Nusselt number is positive for a higher Reynolds number, but negative for all other cases.
- The friction factor decreases with increasing Reynolds number and increases with aspect ratio, but the impact of volume fraction is negligible. Overall, the study provides valuable insights into the behavior of a deep cavity fixed by a rectangular channel under forced convection, and could be useful in designing and optimizing such systems.

## Data Availability

All data used in this manuscript have been presented within the manuscript. No data are hidden or restricted.
